# Bone metastases from differentiated thyroid cancer: characteristics and prognostic factors in a multicenter series

**DOI:** 10.1530/ETJ-23-0086

**Published:** 2023-08-23

**Authors:** Ana Piñar-Gutiérrez, Ana R Romero-Lluch, Suset Dueñas-Disotuar, Irene de Lara-Rodríguez, María Ángeles Gálvez-Moreno, Tomás Martín-Hernández, Jorge García-Alemán, Guillermo Martínez-de Pinillos, Elena Navarro-González

**Affiliations:** 1 UGC Endocrinología y Nutrición, Hospital Universitario Virgen del Rocío, Sevilla, España; 2 Servicio de Endocrinología, Hospital Universitario Reina Sofía, Córdoba, España; 3 Servicio de Endocrinología, Hospital Universitario Virgen Macarena, Sevilla, España; 4 Servicio de Endocrinología, Hospital Universitario Virgen de la Victoria, Málaga, España; 5 Servicio de Endocrinología, Hospital Universitario Virgen de Valme, Sevilla, España

**Keywords:** thyroid cancer, bone metastases, survival, skeletal-related events, radioiodine, multikinase inhibitors, antiresorptive agents

## Abstract

**Objective:**

The aim of this study is to describe the characteristics, survival and prognostic factors of a cohort of patients with bone metastases (BMs) from differentiated thyroid carcinoma (DTC).

**Methods:**

This was a multicenter retrospective observational study including patients diagnosed with BMs from DTC between 1980 and 2021. A Cox regression was performed to study prognostic factors for 5- and 10-year survival. Kaplan–Meier and log-rank tests were performed for the survival analysis and comparison between groups.

**Results:**

Sixty-three patients were evaluated. Median follow-up from BM diagnosis was 35 (15–68) months. About 30 (48.4%) patients presented with synchronous BMs. Regarding histology, 38 (60.3%) had the papillary variant. BMs were multiple in 32 (50.8%) patients. The most frequent location was the spine (60.3%). Other metastases were present in 77.8%, mainly pulmonary (69.8%). Concerning treatment, 54 (85.9%) patients received I131, with BM uptake in 31 (49.2%) and 25 (39.7%) received treatment with multikinase inhibitors. Regarding complications, 34 (54%) patients had skeletal-related events, 34 (54%) died and 5- and 10-year overall survival was 42.4% and 20.4%, respectively. Significant prognostic factors in the multivariate analysis were the presence of lymph node involvement (hazard ratio (HR): 2.916; 95% confidence interval (CI): 1.013–8.391;* P = *0.047) and treatment with I131 (HR 0.214 (95% CI 0.069–0.665);* P = *0.008) at 5 years, the presence of other metastases (HR 6.844. 95% CI 1.017–46.05;* P = *0.048) and treatment with I131 (HR 0.23 (95% CI 0.058–0.913);* P = *0.037) at 10 years.

**Conclusions:**

Our study reflects the management of patients with bone metastases from differentiated thyroid carcinoma in real clinical practice in several centers in southern Spain. Overall survival at 5 and 10 years was lower in patients who were not treated with I131, had nodal involvement and/or had other metastases.

## Introduction

Bone metastases (BMs) are infrequent in differentiated thyroid carcinoma (DTC), occurring in 2–13% of the cases. It has been estimated that BMs occur in 7–28% of follicular carcinomas and 1.4–7% of papillary carcinomas, although data vary considerably in published studies (1). Bones are the second most frequent site of DTC metastases after lung metastases (1). BMs worsen the prognosis of patients with DTC, with published 5- and 10-year survival rates of 61% and 27%, respectively (2). The described factors associated with a better prognosis in these patients are a low volume of BMs (3), single BM (4), early detection of BMs (3), absence of other metastases (5), treatment with I131 (5, 6), I131 uptake by BMs (3, 5) and surgical treatment followed by the treatment with I131 (4). Factors associated with worse prognosis are older age (3, 7) and male sex (7).

BMs are usually clinically silent at onset. Their most frequent clinical presentation is pain followed by fractures (8). Skeletal-related events (SREs) (fractures, spinal cord compression, hypercalcemia of malignancy, need for surgery due to pain and need for radiotherapy due to pain) occur in 55–78% of DTC patients with BMs (9), worsen patient quality of life (10) and have been associated with increased mortality (7).

Treatment may be aimed at palliating the symptoms, preserving or restoring the anatomy and/or modifying the disease (1). The mainstay for pain management is rehabilitation, glucocorticoids and opioids when necessary. Preservation of structures is achieved through surgery, radiotherapy, embolization and ablative therapy. Finally, the two most relevant disease-modifying therapies currently are treatment with I131 and tyrosine kinase inhibitors (TKIs). However, in up to 50% of cases, BMs have the absence of radioiodine uptake (11), and data on the effectiveness of TKIs are limited in this clinical entity (12). In parallel to these treatments, the role of antiresorptive drugs is fundamental in reducing or delaying SRE (9), but their recommendation is currently based on weak evidence. More specific guidelines on the treatment of these patients are therefore needed to improve patient survival and quality of life. Further observational comparative studies on the safety and efficacy of specific treatments for DTC BMs are essential for the development of therapies.

The main objective of our study was to describe the characteristics, management and outcomes in real clinical practice and overall survival in a cohort of patients with BMs from DTC. The secondary objective was to identify prognostic factors associated with overall mortality in this group of patients.

## Methods

### Study design

This was a retrospective observational multicenter study including adult patients with BMs from DTC treated in six hospitals in Andalusia (Spain) between 1980 and 2021. The study was approved by the ethics committee of Virgen del Rocío University Hospital.

### Variables collected

The variables collected were sex, age (at DTC and BM diagnosis), follow-up time (using BM diagnosis as time zero), BM diagnosis (in the initial evaluation of DTC vs during its evolution), histologic variant (aggressive variants were considered to be tall cell and solid/trabecular pattern variants), ultrasensitive thyroglobulin level at DTC diagnosis, stage according to the eighth edition of the TNM (13), characteristics of the BM (single or multiple, number and location), presence of metastases in other locations (lung, brain, mediastinum, adrenal glands and liver), treatments performed (antiresorptives, corticosteroids, I131 – including doses before and after BM diagnosis and uptake by BM – radiotherapy, surgery and TKIs) and their patterns and the presence of SRE (fractures, spinal cord compression, hypercalcemia of malignancy, need for surgery due to pain and need for radiotherapy due to pain) and death.

### Statistical analysis

The statistical analysis was conducted using Statistical Package for Social Sciences (SPSS®) version 25 and R tool for Windows. The descriptive analysis was performed by obtaining the median and the quartiles for quantitative variables (expressed as P50(P25–P75)) and frequency for qualitative variables (expressed as *n* (%)). The Chi-square test was used for the comparison of proportions. To study risk factors for mortality at 5 and 10 years, a univariate analysis was performed using Cox regression and subsequently a multivariate analysis with the variables that obtained a *P* < 0.1. The overall survival analysis was performed using the Kaplan–Meier method, and the log-rank test was used to compare survival between groups. A *P*-value less than 0.05 was considered statistically significant.

## Results

### Demographic and clinical characteristics

The sample comprised 63 patients.

The baseline characteristics of the patients are shown in Table 1. The follow-up time was 35 (15–68) months, with a minimum of 12.8 and a maximum of 515.8. The age at BM diagnosis was 68 (57–73) years and 32 (50.8%) patients had metachronous BMs. The most frequent histologic variant was papillary (60.3%), 42.1% of which were aggressive variants. Of these patients, 60.3% had stage T3 or T4 disease, 41.3% had lymph node involvement, 50.8% had multiple BMs and 77.8% had metastases in other locations.

**Table 1:** Description of baseline characteristics. Quantitative variables are expressed as P50 (P25–P75) and qualitative variables as *n* (%).

Variable	Result
Male sex	27 (42.8%)
Age at diagnosis	
*DTC*	62 (52–71)
*BM*	68 (57–73)
Follow-up time (months)	35 (15–68)
Metachronous BMs	
Time between DTC diagnosis and BM diagnosis in metachronous (months)	32 (50.8%)
	80 (25–201)
Follicular carcinoma	24 (38.1%)
Papillary carcinoma	38 (60.3%)
Aggressive variants^a^	16 (42.1%)
Oncocytic carcinoma	1 (1.5%)
Ultrasensitive thyroglobulin at diagnosis (ng/mL)	1000 (195–7743)
T	
X	9 (14.3%)
1	9 (14.3%)
2	7 (11.1%)
3	16 (25.4%)
4	9 (34.9%)
N1	26 (41.3%)
Multiple BMs	32 (50.8%)
Number of BMs	2 (1–4)
BM location	
Cranial	11 (17.5%)
Spinal column	38 (60.3%)
Rib cage	21 (33.3%)
Extremities	25 (39.7%)
Pelvis	20 (31.7%)
Metastases in other locations	49 (77.8%)
Pulmonary	44 (69.8%)
Cerebral	6 (9.5%)
Mediastinal	8 (12.7%)
Adrenal	3 (4.8%)
Hepatic	6 (9.5%)

^a^Aggressive variants: tall cell variants and solid-trabecular pattern.

The patients with synchronous BMs at DTC diagnosis had fewer metastases in other locations (*P = *0.01), had higher I131 uptake (*P* < 0.001), received higher doses of I131 after BM diagnosis (400 (250–570) vs 0 (0-112) mCi; *P* < 0.001) – due to their increased uptake of I131 – and received TKI treatment less frequently (*P = *0.034) than those with metachronous BMs. There were no other differences between the two groups.

### Treatments and events

The treatments performed and the results are shown in Table 2. Fifty-four (85.9%) patients received treatment with I131, of whom 31 (49.2%) had radioactive iodine-avid BM. Twenty-one (33.3%) received antiresorptive treatment, 22 (34.9%) treatment with corticoids and 25 (39.7%) treatment with TKIs. Regarding events, 34 (54%) presented SRE, highlighting fracture, which occurred in 16 (25.4%) patients. Thirty-four (54%) died.

**Table 2:** Treatments and events.

Variable	Result^a^
Total thyroidectomy	54 (85.9%)
Antiresorptive treatment	21 (33.3%)
Zoledronic 4 mg i.v. monthly	16 (76.1%)
Denosumab 60 mg s.c. semiannually	3 (14.2%)
Alendronic acid 70 mg v.o. weekly	2 (9.5%)
Corticosteroids	22 (34.9%)
I131	
Treated	54 (85.9%)
BM uptake	31 (49.2%)
Total I131 dose (mCi)	350 (157–535)
Post-diagnostic total I131 dose BM	150 (0–425)
BM surgery	23 (36.5%)
BM radiotherapy	32 (50.8%)
TKI treatment	25 (39.7%)
Lenvatinib first line	8 (32%)
Sorafenib first line	7 (28%)
Lenvatinib second line	9 (36%)
Axitinib second line	1 (4%)
SRE	34 (54%)
Fracture	16 (25.4%)
Spinal cord compression	8 (12.7%)
Surgery for pain	7 (11.1%)
RT for pain	20 (31.7%)
Hypercalcemia of malignancy	0 (0%)
Death	34 (54%)
Age	75 (60.25–78.25)
Follow-up time between DTC diagnosis and death (months)	72 (43–149)
Follow-up time between diagnosis of BM and death (months)	32 (11–52)

^a^Quantitative variables are expressed as P50 (P25–P75) and qualitative variables as *n* (%).

CI, Confidence interval; HR, hazard ratio.

There were no statistically significant differences when comparing the presence of lymph node metastasis (N1), more advanced tumor (T) stages (*P = *0.423), presence of multiple bone metastases (*P = *0.304), presence of metastases in other locations (*P = *1) or presence of pulmonary metastases (0.823) between patients who received treatment with I131 and those who did not (X2 test).

Patients with multiple BM did not receive more treatment with I131 (*P = *0.304), antiresorptives (*P = *0.212), corticosteroids (*P = *0.135), surgery (*P = *0.49), radiotherapy (*P = *0.707) or TKIs (*P = *0.719) than patients with a single BM. The percentage of SRE in patients with a single BM was 65.6% vs 41.9% in patients with multiple BMs, with a result close to significance (*P = *0.059).

Of the 21 patients who received antiresorptive therapy, in 18 (85.7%) it was prescribed after the patient experienced an SRE. Of the three who received these drugs preventively, two had not had an SRE. There were no significant differences between the patients who received this treatment and those who did not, but the results were close to significance in terms of spinal cord compression (23.8% vs 7.1%, *P = *0.061). Of the eight patients treated with TKIs and antiresorptive agents, 2 (25%) presented mandibular osteonecrosis in the context of treatment with zoledronic acid and TKIs (sorafenib and axitinib in each case). One of them required surgery.

## Determinants of bone metastasis

### Prognostic factors for mortality

The results of the univariate analysis of risk factors for 5-year mortality are shown in Table 3. The variables that were statistically significant were diagnosis of BMs at DTC evaluation (hazard ratio (HR): 2.51; 95% confidence interval (CI:) 1.09–5.78; *P = *0.031), metastases in other locations (HR: 10.939; 95% CI: 1.477–80.919); *P = *0.019, lung metastases (HR: 3.593; 95% CI: 1.234–10.462; *P = *0. 019), corticosteroid treatment (HR: 2.311; 95% CI: 1.062–5.027); *P = *0.035, I131 treatment (HR: 0.359; 95% CI: 0.165–0.944; *P = *0.037), I131 dose after BM diagnosis (HR: 0.995; 95% CI: 0.992–0.998); *P = *0.003 and I131 uptake by BMs (HR: 0.22; 95% CI: 0.08–0.6; *P = *0.003).

**Table 3:** Results of the univariate analysis of risk factors associated with overall mortality at 5-year follow-up.

Variable	HR	95% CI	*P*
Male sex	0.818	(0.37–1.808)	0.619
Age at DTC diagnostic	0.996	(0.965–1.028)	0.798
>65 years	0.78	(0.36–1.7)	0.5
Age at BM diagnostic	1.025	(0.983–1.07)	0.244
Metachronous BMs	2.51	(1.09–5.78)	0.031^a^
Follicular carcinoma	0.878	(0.388–1.987)	0.754
Thyroglobulin at diagnosis (ng/mL)	1	(1–1)	0.573
Tumor stage (1–2 vs 3–4)	2.888	(0.844–9.885)	0.091^b^
Nodal metastasis (N1)	2.464	(0.952–6.337)	0.063^b^
Multiple BMs	0.747	(0.345–1.617)	0.459
Number of BMs	0.933	(0.754–1.153)	0.521
BM location			
Cranial	0.362	(0.108–1.212)	0.099^b^
Spinal column	1.262	(0.57–2.794)	0.566
Rib cage	1.159	(0.525–2.557)	0.715
Limbs	0.586	(0.254–1.35)	0.209
Pelvis	0.94	(0.405–2.178)	0.884
Metastases in other locations	10.934	(1.477–80.919)	0.019^a^
Pulmonary	3.593	(1.234–10.462)	0.019^a^
Cerebral	2.042	(0.6–6.954)	0.253
Mediastinal	1.986	(0.79–4.988)	0.144
Adrenal	0.557	(0.075–4.127)	0.567
Hepatic	1.192	(0.356–3.991)	0.776
Anti-resorptive treatment	0.749	(0.325–1.725)	0.497
Corticosteroid treatment	2.311	(1.062–5.027)	0.035^a^
I131 treatment	0.395	(0.165–0.944)	0.037^a^
Total dose of I131	0.999	(0.997–1.001)	0.489
I131 dose after BM diagnosis	0.995	(0.992–0.998)	0.003^a^
I131 uptake by BMs	0.22	(0.08–0.6)	0.003^a^
BM surgery	0.556	(0.241–1.286)	0.17
BM radiotherapy	0.985	(0.45–2.157)	0.97
TKI treatment	1.56	(0.71–3.4)	0.3
SRE	1.09	(0.49–2.42)	0.8
Fractures	0.667	(0.277–1.605)	0.366
Spinal cord compression	1.223	(0.367–4.079)	0.743
Surgery for pain	1.118	(0.218–5.733)	0.894
Radiation therapy for pain	2.497	(0.79–7.897)	0.119

^a^*P* < 0.05; ^b^*P* < 0.1.

CI, confidence interval; HR, hazard ratio.

When studying combinations of treatments (I131 + TKIs, I131 + antiresorptive treatment, TKIs + antiresorptive treatment and RT + antiresorptive treatment) and their association with mortality, the groups obtained were of insufficient size to perform Cox regression. Therefore, a chi-square test was performed. None of the results reached statistical significance.

The variables that were statistically significant in the 5-year multivariate analysis were the presence of lymphatic involvement (N1) (HR: 2.916; 95% CI: 1.013–8.391; *P = *0.047) and treatment with I131 (HR: 0.214; 95% CI: 0.069–0.665; *P = *0.008). The presence of pulmonary metastases yielded a result close to significance (HR: 2.784; 95% CI: 0.872–8.884; *P = *0.084).

The results of the univariate analysis of risk factors for 10-year mortality are shown in Table 4. The statistically significant variables were metastases in other locations (HR: 4.306; 95% CI: 1.303–14.225; *P = *0.017), corticosteroid treatment (HR: 2.417; 95% CI: 1.18–4.95; *P = *0.016), I131 dose after BM diagnosis (HR: 0.997; 95% CI: 0.995–0.999; *P = *0.009) and I131 uptake by BMs (HR: 0.35; 95% CI: 0.15–0.8; *P = *0.012).

**Table 4:** Results of the univariate analysis of risk factors associated with overall mortality at 10-year follow-up.

Variable	HR	95% CI	*P*
Male sex	1.014	(0.48–2.142)	0.970
Age at DTC diagnostic	1.006	(0.976–1.036)	0.716
*>*65 years	0.88	(0.43–1.79)	0.7
Age at BM diagnostic	1.03	(0.99–1.072)	0.148
Metachronous BMs	2.07	(0.99–4.34)	0.053^b^
Follicular carcinoma	0.855	(0.406–1.8)	0.679
Thyroglobulin at diagnosis (ng/mL)	1	(1–1)	0.514
Tumor stage (1–2 vs 3–4)	2.798	(0.949–8.249)	0.062^b^
Nodal metastasis (N1)	2.078	(0.875–4.933)	0.097^b^
Multiple BMs	0.518	(0.25–1.071)	0.076^b^
Number of BMs	0.988	(0.821–1.191)	0.903
BM location			
Cranial	0.605	(0.245–1.493)	0.276
Spinal column	1.452	(0.694–3.308)	0.322
Rib cage	1.119	(0.542–2.331)	0.761
Limbs	0.758	(0.361–1.589)	0.463
Pelvis	0.925	(0.422–2.027)	0.845
Metastases in other locations	4.306	(1.303–14.225)	0.017^a^
Pulmonary	2.251	(0.966–5.245)	0.06^b^
Cerebral	2.042	(0.6–6.954)	0.253
Mediastinal	2.219	(0.938–5.245)	0.069^b^
Adrenal	0.34	(0.046–2.532)	0.292
Hepatic	1.558	(0.537–4.517)	0.415
Anti-resorptive treatment	0.741	(0.348–1.581)	0.439
Corticosteroid treatment	2.417	(1.18–4.95)	0.016^a^
I131 treatment	0.477	(0.204–1.112)	0.087^b^
Total dose of I131	0.999	(0.998–1.001)	0.511
I131 dose after BM diagnosis	0.997	(0.995–0.999)	0.009^a^
I131 uptake by BMs	0.35	(0.15–0.8)	0.012^a^
BM surgery	0.613	(0.287–1.308)	0.206
BM radiotherapy	1.041	(0.5–2.166)	0.915
TKI treatment	1.35	(0.66–2.75)	0.4
SRE	1.31	(0.61–2.79)	0.5
Fractures	0.793	(0.368–1.705)	0.552
Spinal cord compression	2.201	(0.766–5.327)	0.155
Surgery for pain	0.877	(0.181–4.237)	0.87
Radiation therapy for pain	2.465	(0.883–6.885)	0.085^b^

^a^*P* < 0.05; ^b^*P* < 0.1.

CI, confidence interval; HR, hazard ratio.

The variables that were statistically significant in the 10-year multivariate analysis were the presence of metastases in other locations (HR: 6.844; 95% CI: 1.017–46.05; *P = *0.048) and treatment with I131 (HR: 0.23; 95% CI: 0.058–0.913; *P = *0.037). The presence of lymphatic involvement (N1) scored close to significance (HR: 3.101; 95% CI: 0.822–11.702; *P = *0.095).

### Survival analysis

**Figure 1:**
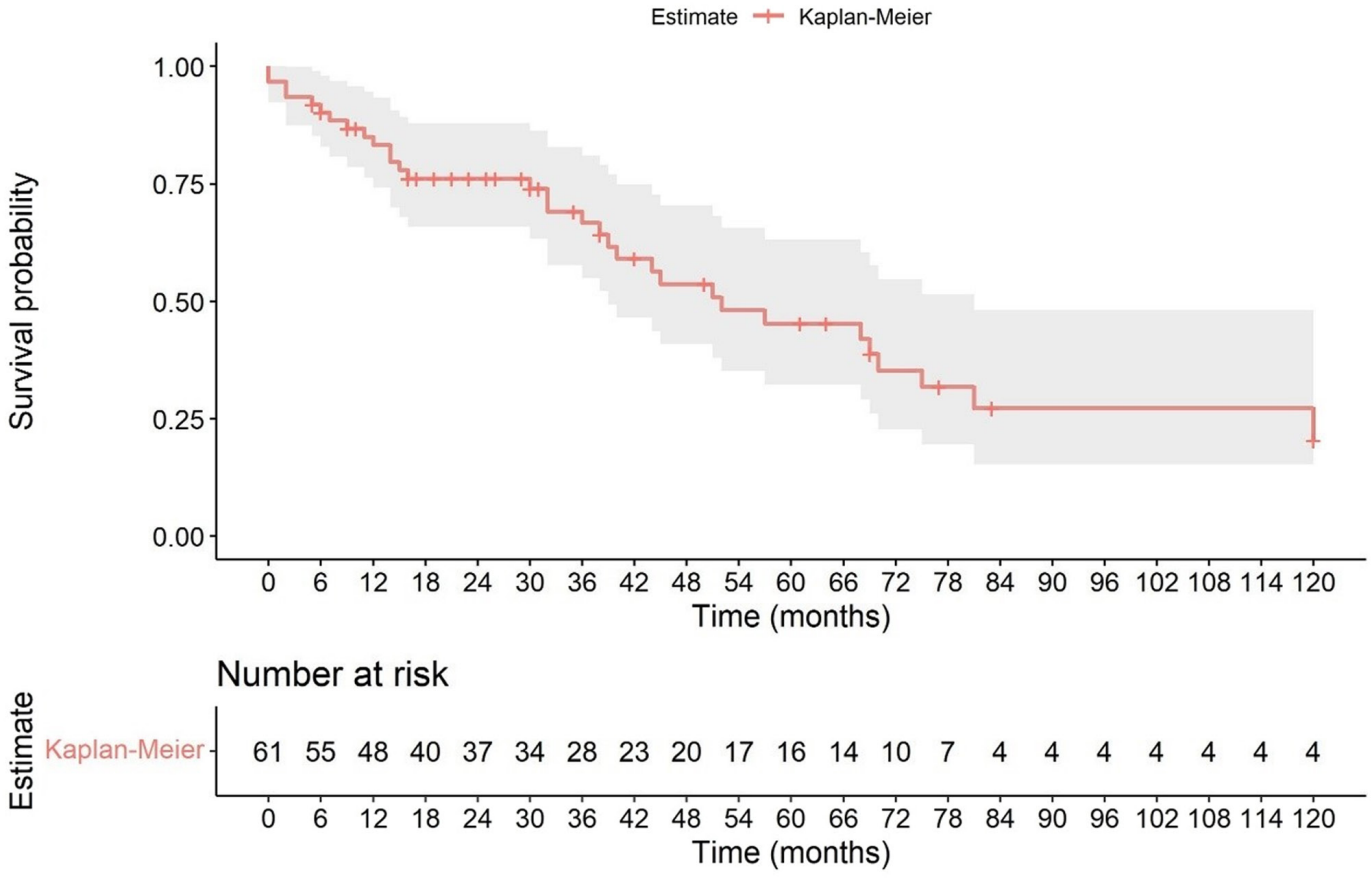
Overall survival.

Overall survival at 3 years was 64.2%, at 5 years 42.4% and at 10 years 20.4% (Fig. 1).

**Figure 2:**
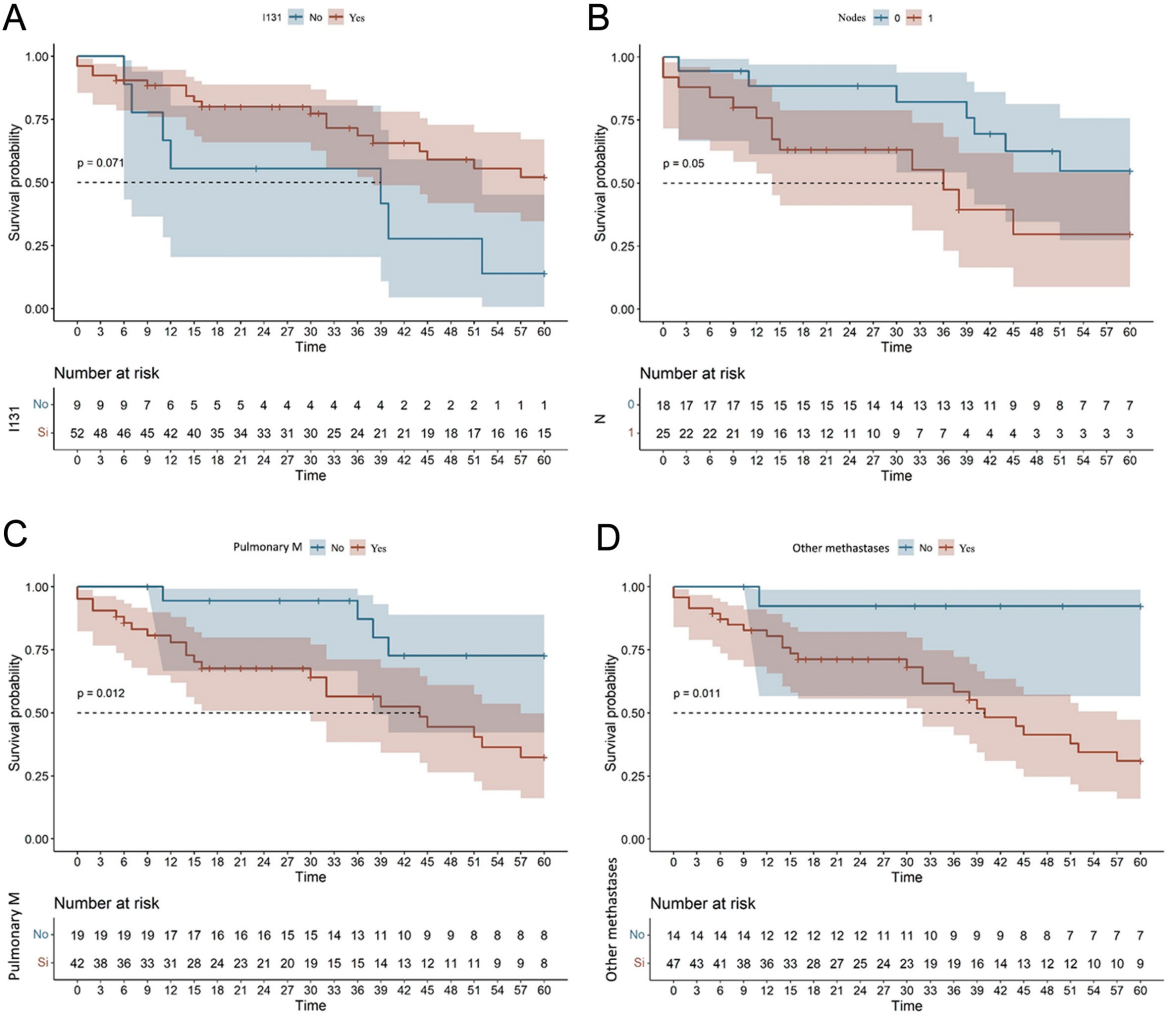
Survival plots at 5 years according to whether patients received I131 treatment or not (A); the presence of nodal disease (N1) at 5 years (B); the presence of pulmonary metastases at 5 years (C); and the presence of other metastases at 10 years (D).

The overall survival plots according to the most relevant factors that reached statistical significance in the multivariate analysis are shown in Fig. 2. When performing a log-rank analysis at 5 years, there were significant differences between the groups with and without lymph node involvement (*P = *0.05) and with and without pulmonary metastases (*P = *0.012), and differences approached the significance between the groups that received treatment with I131 and those that did not (*P = *0.071). When analyzing 10-year survival, there were significant differences between the group with other metastases and the group without metastases (*P = *0.013).

## Discussion

The studies published to date on BMs in patients with thyroid cancer are observational and retrospective. In the last 5 years, seven studies stand out, six of which are based on a series of between 64 and 178 patients (2, 6, 14, 15, 16, 17) and the remaining one includes 1173 patients from a US national database (Medicare) (7). All of them included patients with DTC, although in the study by Choksi *et al.* (7), patients with medullary carcinoma were also included. Only two of the studies based on a series of patients were multicenter (6, 14), and the follow-up time ranged from 48 months (6) to 11 years (16).

Our study analyzed the characteristics of a cohort of 63 patients with BMs from DTC treated in different centers in Andalusia (Spain). The overall survival at 5 and 10 years was 42.4% and 20.4%, respectively. Furthermore, our study supported I131 treatment as a prognostic factor for survival, as described in other studies (2, 5, 6). The risk factors for mortality in the multivariate analysis were the presence of lymphatic involvement and the presence of other metastases.

Regarding patient characteristics, both age and histological variants were similar to those published by Mazziotti *et al.* (6), a study on BMs from DTC comparable to ours because it was multicenter and performed in another Mediterranean country. The extent of disease according to TNM classification was similar in terms of tumor size, but they found a lower percentage of patients classified as lymph node metastases (29.8% vs 41.3%) and fewer patients with pulmonary metastases (51.7% vs 69.8%). In their study, 50.3% of the patients presented synchronous BMs, similar to our results (48.4%), although the time between the diagnosis of DTC and BMs in those with metachronous BMs was not similar (median of 60 months vs 80 months). On the other hand, the percentage of patients with multiple BMs was higher than ours (66.4% vs 50.8%) but the median number of BMs was two, as in our study. The most frequent location was the spine, as described in other series (18).

Regarding the treatments used in our study, the percentage of patients who received treatment with I131 was also similar to that obtained by Mazziotti *et al.* (6), although their patients received a higher median total dose of I131 (563 vs 350mCi). The use of antiresorptive therapy was lower in their study (22.4% vs 33.3%). The use of this therapy is low in both studies in view of their expected benefit and the general recommendations in the guidelines (9, 19). The fact that 85.7% received antiresorptive drugs after SRE is also a result that could be improved in our daily clinical practice.

It should be noted that 25% of the patients who received combined treatment with TKIs and antiresorptive drugs (zoledronic acid) presented osteonecrosis of the jaw. In December 2010, the European Medicines Agency issued safety warnings about ONJ risk in patients receiving sunitinib or bevacizumab. To date, there is little published data on the combination of these two treatments and none in patients with DTC. Moreover, studies on this subject are usually small and retrospective. They are also limited by the fact that most of the patients studied have a short survival and therefore a short treatment with bisphosphonates. In a study conducted in 76 patients with renal carcinoma and bone metastases, the combination of both treatments not only had an impact on patient survival but also increased the risk of osteonecrosis of the jaw (20). Further studies are therefore needed to evaluate their safety.

Continuing with TKIs, most real-life studies and clinical trials report worse response of BMs to these drugs compared to other metastases such as lung metastases (21). It should also be kept in mind that they can produce fistulas and bleeding and care should be taken with lesions near the trachea and/or esophagus, although this has not been described in BMs to our knowledge (22). Bone fractures have also been suggested as a contraindication for treatment with sorafenib (22).

Finally, SRE were less frequent in the study by Mazziotti *et al.* (37.1% vs 54%) than in our study, although they reported similar rates of fracture (24.5% vs 25.4%) and spinal cord compression (11.9% vs 12.7%). Their percentage of deaths was also lower than ours (27.3% vs 54%), despite a longer median follow-up time in months (48 vs 35). This may be explained by their lower percentage of patients with N1 and pulmonary metastases, which was associated with lower survival in our study. It was not possible to study whether treatment with antiresorptive drugs was protective for SRE, since only three patients received these treatments preventively and not after having had an SRE.

Regarding survival, the first data were published in the 1980s, with a 10-year survival range of 13–21% (23, 24, 25). Subsequent studies have shown similar data: Durante *et al.* (26) found a 10-year survival rate of 25% in patients with BMs and 13% in patients with BMs and pulmonary metastases; Jannin *et al.* (14) found survival rates of 70% and 26%; and in the study by Wu *et al.* (2) these rates were 61% and 27% at 5 and 10 years, respectively. However, there are studies with better survival data. For example, in the study by Matta-Coelho (17), the 5- and 10-year survival was 60% and 50%, respectively. In addition, Pittas *et al.* (27) reported survival rates of 53% and 35% at 5 and 10 years; Choi *et al.* (15) reported 77.1% and 46.6%, respectively; and Slook *et al.* (16) reported 45.3% survival at 10 years. These variations are due to the different characteristics of the studies performed and patients included as well as the treatments administered.

Treatment with I131 was a protective factor for mortality in our cohort. This result is similar to the findings of other published studies (2, 5, 6). The importance of giving RAI to those patients is also reinforced by the fact that, in our cohort, patients with I131 treatment were similar at baseline to those who didn't receive it (similar data for TNM stage, metastases in other locations and pulmonary metastases, which were the factors associated with mortality in our study). The uptake of I131 by BMs was also significant as a protective factor in the univariate analysis, a result consistent with those of other authors (6, 14, 17, 28, 29). Regarding the combination of I131 with other treatments, this was not shown to improve survival in our cohort, contrary to the results of other studies (2, 4, 30, 31, 32, 33, 34, 35). Corticosteroid use was associated with an increased risk of death in univariate analysis. Nonetheless, this is likely because patients with worse clinical status received more glucocorticoids.

The extent of disease according to TNM classification also appears to be related to prognosis. Specifically, the T staging result from the univariate analysis approached significance at both 5 years (*P = *0.091) and 10 years (*P = *0.062). This was the only factor related to mortality in the study published in 1999 by Lin *et al.* (36). In the studies by Mazziotti *et al.* (6) and Jannin *et al.* (14), a T4 stage was associated with higher mortality in the univariate analysis, and in the study by Slook *et al.* (16), significance was also reached in the univariate analysis for patients with stage T3 or T4. N1 was significant in the multivariate analysis at 5 years (*P = *0.047) and close to significance at 10 years (*P = *0.095), similar to that published by other authors (16).

Finally, in our study, the presence of pulmonary metastases obtained a result close to significance in the 5-year multivariate analysis (*P = *0.084), and the presence of metastases other than BMs was a factor related to lower 10-year survival (*P = *0.048). These have previously been described as factors associated with mortality in other studies (4). The result for multiple BMs was close to significance in the 10-year multivariate analysis (*P = *0.076). This is congruent with the results of other studies (4, 37, 38).

Metachronous BMs were also associated with higher mortality in the univariate analysis but not in the multivariate analysis. This is in agreement with the results of Mazziotti *et al.* (6) and can be explained precisely because patients with metachronous BMs presented more metastases in other locations (4), received lower doses of I131 after BM diagnosis and presented lower I131 uptake, which is related to lower survival (2, 29). Patients with synchronous BMs received higher doses of iodine mainly because they had higher uptake. This may also be influenced because when BMs are diagnosed together with DTC, the initial doses of I131 are already initially higher simply because of having metastases. Furthermore, it may also happen that patients presenting with metachronous BMs have a more rapidly progressive disease refractory to I131 treatment and therefore are not given iodine later on. In relation to age, the BM was not associated with worse survival. In the study by Slook *et al.* (16), age >45 years was associated with higher mortality; in Wu *et al.* (2), age <55 years was associated with better survival; and in Choksi *et al.* (7), age >65 years was associated with worse survival. In our cohort, the interquartile range was 62 (52–71) years for DTC diagnosis and 68 (57–73) years for BM diagnosis, which makes it difficult together with the sample size to reach statistical significance for those age ranges. Other variables that have been found to be significant in other studies but not in ours are SRE (6, 7, 39), spine location (16), surgery (4) and treatment with zoledronic acid (35).

The limitations of the study, in addition to those previously mentioned, are its observational and retrospective nature and its relatively small sample size, which made it difficult to obtain statistically significant results. The lack of a joint protocol for the care of these patients in the different centers studied could also lead to bias in the interpretation of certain results, since different treatments could have been applied to different patient profiles. Another limitation is that we included cases followed from 1980 to 2021 with different histopathological and TNM classifications being used throughout this time period, although the TNM classification was adapted in all patients so that it was always collected according to the eighth edition. The strengths of our study lie in its multicentricity (including hospitals of different levels in the same health area and therefore faithfully representing real clinical practice in the approach to this disease) and in the long follow-up time, with a median of 35 months and a maximum of 515 months.

## Conclusions

Our study reflects the management of patients with BMs from DTC in real clinical practice in several centers in southern Spain. The use of antiresorptive drugs was lower than recommended based on current evidence on their preventive role in SRE, which were present in 54% of our patients. Overall survival at 5 and 10 years was lower in patients who were not treated with I131, had nodal involvement and/or had other metastases.

Given the low frequency of BMs from DTC, multicenter studies involving a larger number of patients will help to improve our understanding of this disease and the treatment of these patients. The implementation of unified management protocols would also be beneficial both to improve the quality of the studies performed and to provide patients with better care.

## Declaration of interest

The authors declare that there is no conflict of interest that could be perceived as prejudicing the impartiality of the research reported.

## Funding

This research did not receive any specific grant from any funding agency in the public, commercial or not-for-profit sector.

## Author contribution statement

All authors collected the data. A P-G analyzed the data and wrote the first draft of the manuscript. E G-N conceived the study. All authors reviewed and corrected the manuscript.
